# Unlocking Hope: Therapeutic Advances and Approaches in Modulating the Wnt Pathway for Neurodegenerative Diseases

**DOI:** 10.1007/s12035-024-04462-4

**Published:** 2024-09-23

**Authors:** Navid Faraji, Negar Ebadpour, Mohammad Abavisani, Ali Gorji

**Affiliations:** 1 Student Research Committee, Mashhad University of Medical Sciences, Mashhad, Iran; 2 Immunology Research Center, Mashhad University of Medical Sciences, Mashhad, Iran; 3 Neuroscience Research Center, Mashhad University of Medical Sciences, Mashhad, Iran; 4 Epilepsy Research Center, Münster University, Münster, Germany; 5 Shefa Neuroscience Research Center, Khatam Alanbia Hospital, Tehran, Iran; 6 Neurosurgery Department, Münster University, Münster, Germany

**Keywords:** Alzheimer’s disease, Molecular targeted therapy, Neurodegeneration, Parkinson’s disease, Wnt signaling pathway

## Abstract

Neurodegenerative diseases (NDs) are conditions characterized by sensory, motor, and cognitive impairments due to alterations in the structure and function of neurons in the central nervous system (CNS). Despite their widespread occurrence, the exact causes of NDs remain largely elusive, and existing treatments fall short in efficacy. The Wnt signaling pathway is an emerging molecular pathway that has been linked to the development and progression of various NDs. Wnt signaling governs numerous cellular processes, such as survival, polarity, proliferation, differentiation, migration, and fate specification, via a complex network of proteins. In the adult CNS, Wnt signaling regulates synaptic transmission, plasticity, memory formation, neurogenesis, neuroprotection, and neuroinflammation, all essential for maintaining neuronal function and integrity. Dysregulation of both canonical and non-canonical Wnt signaling pathways contributes to neurodegeneration through various mechanisms, such as amyloid-β accumulation, tau protein hyperphosphorylation, dopaminergic neuron degeneration, and synaptic dysfunction, prompting investigations into Wnt modulation as a therapeutic target to restore neuronal function and prevent or delay neurodegenerative processes. Modulating Wnt signaling has the potential to restore neuronal function and impede or postpone neurodegenerative processes, offering a therapeutic approach for targeting NDs. In this article, the current knowledge about how Wnt signaling works in Alzheimer’s disease and Parkinson’s disease is discussed. Our study aims to explore the molecular mechanisms, recent discoveries, and challenges involved in developing Wnt-based therapies.

## Introduction

Neurodegenerative diseases (NDs) are a group of disorders that mainly affect the structure and function of neurons in the central nervous system (CNS), leading to progressive loss of cognitive, motor, and sensory abilities [1]. Disabilities and deaths caused by NDs are substantial. On a global scale, neurological disorders caused almost 10 million fatalities and 349 million disability-adjusted life years in 2019. This is a growing public health risk that is predicted to be worsened by an aging population [2]. Even though a lot of investigations have been done, the cause and progression of NDs are still mostly unknown. There are also no effective treatments or cures available, despite some therapeutic approaches that have been described, such as mitochondria-targeted antioxidant therapy or the application of mitochondrial dynamics modulators, epigenetic modulators, and neural stem cell therapy [3, 4].

The Wnt signaling pathway plays a crucial role in maintaining neuronal health and function. In recent years, the Wnt signaling pathway has emerged as a significant molecular mechanism implicated in the onset and progression of NDs. The Wnt signaling pathway controls a wide range of cellular activities through a complicated and highly conserved protein network [5]. Evidence suggests that the Wnt signaling pathway is essential for regulating neuronal activity and maintaining the integrity of the adult CNS. In addition to controlling neurogenesis, neuroprotection, and neuroinflammation, Wnt signaling controls synaptic transmission, plasticity, and memory formation [6, 7]. Accordingly, dysregulation of Wnt signaling has been linked to various NDs. In these diseases, Wnt signaling is either impaired or overactivated, resulting in altered neuronal morphology, connectivity, and viability. Moreover, other pathogenic factors, like amyloid-β (Aβ), tau, α-synuclein, huntingtin, and TDP-43, which are the primary components of protein aggregates in the brains of ND patients, interact with Wnt signaling [8, 9]. Since NDs may be treatable by restoring neuronal function and preventing or delaying neurodegeneration, regulating Wnt signaling may be a promising therapeutic option. Alzheimer’s disease (AD) and Parkinson’s disease (PD) are among the most common NDs globally, with AD being the leading cause of dementia and PD being one of the most prevalent movement disorders. Here, we reviewed the complex role of Wnt signaling in AD and PD with a specific focus on elucidating the underlying molecular mechanisms. Furthermore, we aim to draw attention to current developments and challenges in using Wnt-based treatments for these NDs. Within the context of NDs, our discussion covers a variety of targets and approaches used to modulate Wnt signaling, highlighting their pros and cons.

## The Wnt Pathway

The Wnt pathway is a highly conserved signaling pathway that was first identified in 1987. Multiple studies conducted over the past few decades have established the crucial function of this route in maintaining tissue homeostasis and facilitating cell proliferation, differentiation, migration, and survival. This applies not only to the early stages of embryonic development and organ formation but also through adulthood [5, 10, 11]. Wnt ligands (Wnts) are intercellular lipid-altered proteins that activate the Wnt pathway by binding to Wnt receptors and co-receptors. Then, activated receptors transmit the signal through an intracellular cascade, such as β-catenin and Jnk signaling, or secondary messengers, such as calcium (Ca^2+^). The activation of genes and responses related to Wnts is the final result of this process [12, 13]. Various studies have revealed that the dysregulation of the Wnt pathway has been implicated in numerous diseases, including various cancers, NDs, cardiovascular disorders, and developmental abnormalities [14–17].

The intracellular cascade based on its reliance on β-catenin is divided into two pathways; (i) β-catenin-dependent (canonical) pathway and (ii) β-catenin-independent (non-canonical) pathway. The non-canonical pathway can be further categorized into two distinct pathways: the plantar cell polarity (Pcp) pathway and the Ca^2+^-dependent pathway. The canonical pathway is better comprehended compared to the non-canonical pathway. Despite the marked differences between the canonical and the non-canonical pathways, they do share certain components, including Wnt ligands, particular receptors, and inhibitors. Wnt1, Wnt2, Wnt3a, Wnt3, Wnt7a, Wnt8b, and Wnt10b are ligands contributed to the canonical pathway, and Wnt4, Wnt5a, and Wnt1 are associated with non-canonical pathways [18]. The canonical pathway involves the binding of Wnts to Frizzled (Fzd) receptors and co-receptors LRP5/6. This interaction stabilizes β-catenin and facilitates its nuclear translocation, where it acts as a transcriptional co-activator regulating Wnt target gene expression [18]. The non-canonical pathway involves the activation of alternative downstream effectors, such as the Pcp and the Ca^2+^-signaling pathways, which modulate cytoskeletal dynamics, cell adhesion, and intracellular calcium levels [12].

**Fig. 1:**
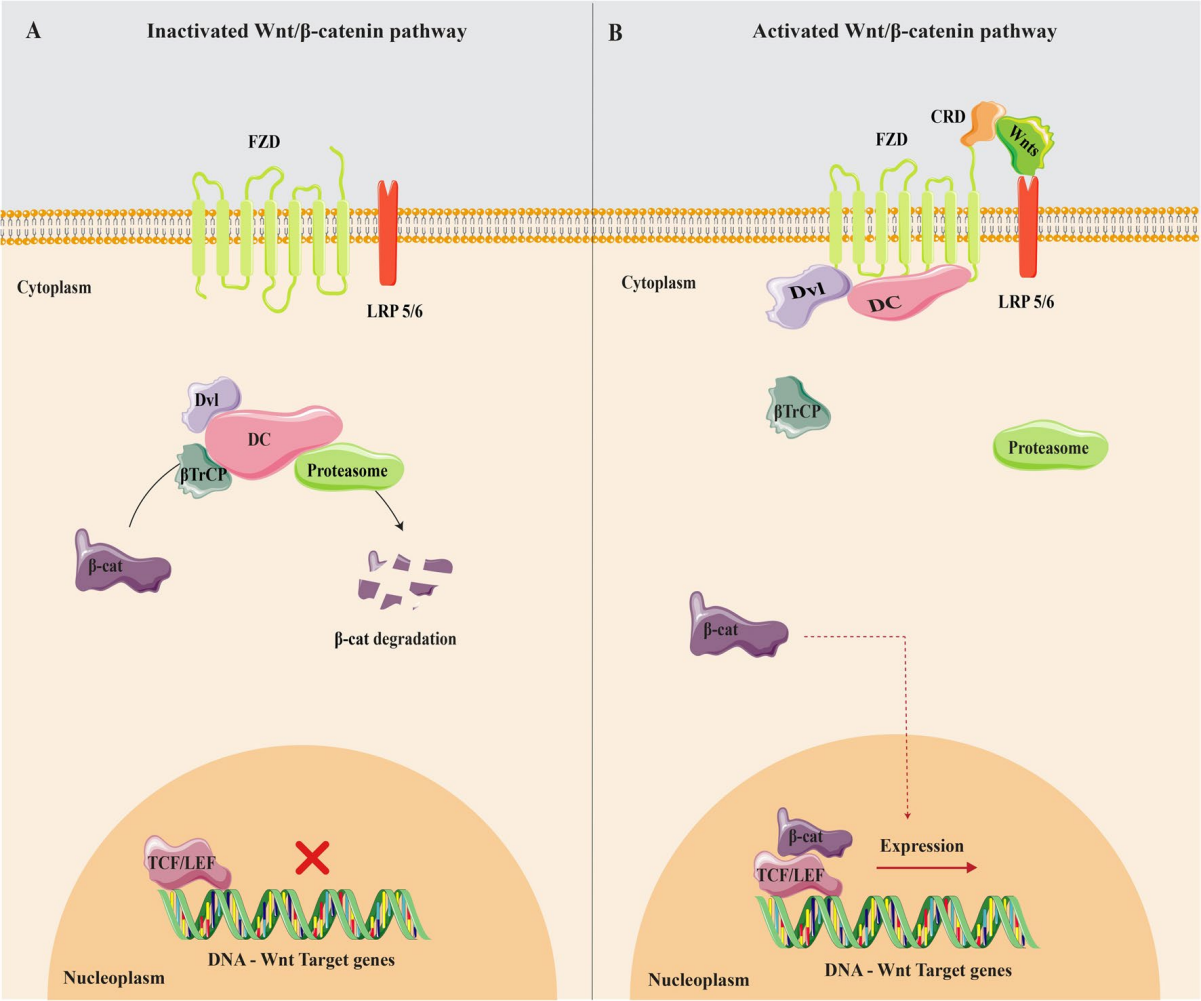
A comparative illustration of inactivated and activated Wnt/β-catenin pathways in cellular signaling. The destruction complex (Dc) consists of Axin, a scaffold protein, APC, GSK3-β, and CK1a, which are two serine-threonine kinases. **A** In the absence of Wnts, β-catenin binds with Axin and gets phosphorylated by CK1a, followed by GSK3-β. The phosphorylated β-catenin subsequently undergoes ubiquitination by E3-ligase β-TrCP2, followed by ubiquitin-dependent destruction mediated by a proteasome. **B** Upon binding of Wnts to receptors, the cytoplasmic domain of Fzd reaches towards the Dishevelled (Dvl) protein which augments the interaction between Axin and LRP. After the inhibition of GSK3-β, the degradation of β-catenin by the proteasome does not take place, resulting in an elevation of the cytoplasmic concentration of β-catenin

The intracellular level of β-catenin which is the main effector of the canonical pathway is controlled by a multimeric complex called the destruction complex (Dc). While the Wnt pathway is not activated, the Dc degrades β-catenin, resulting in a decrease in its cytoplasmic content. This prohibits β-catenin from reaching the nucleus, where it is supposed to induce the expression of Wnt target genes (Fig. 1A). When Wnts bind to the Fzd and LRP5/6 as the co-receptor, the Dc activity is inhibited by the Fzd-Lrp-Dvl complex and β-catenin stabilizes in the cytoplasm. Followed by the increased cytoplasmic level of β-catenin, it is imported to the nucleus where it binds to transcription factors (Tcf/Lef) and activates target gene expression. These genes (e.g., C-myc and Cyclin-19) are responsible for cellular proliferation and differentiation (Fig. 1B) [18–20].

**Fig. 2:**
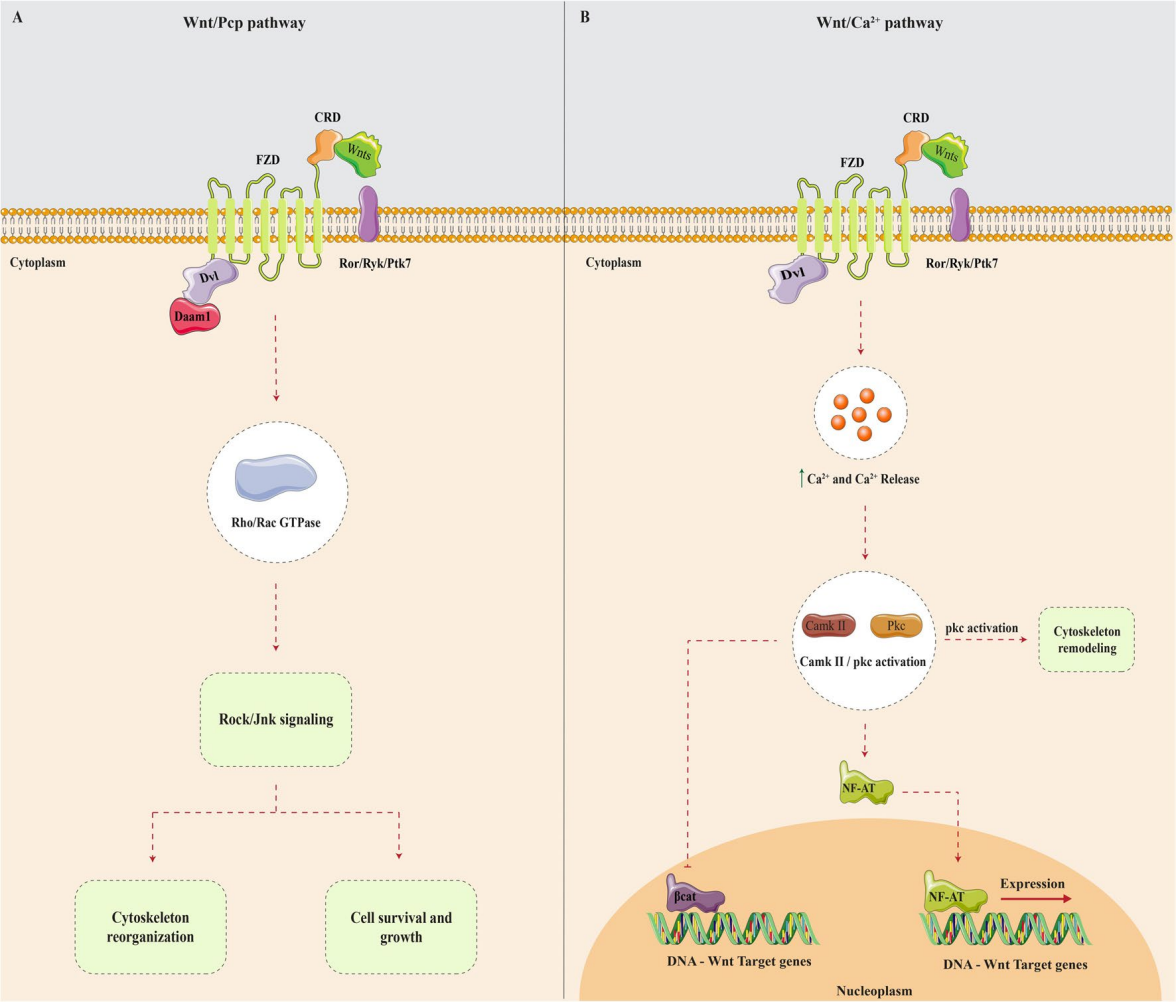
The Wnt/Pcp signaling pathway (**A**) and the Wnt/Ca^2+^ signaling pathway (**B**) are depicted here. **A** Wnt ligands bind to Fzd, and then, via Ror/Ryk/Ptk7 receptors, activate Dvl and Daam1. Daam1 activates Rho/Rac GTPase, which triggers Rock/Jnk signaling. Rock/Jnk signaling induces cytoskeleton reorganization, resulting in cell survival and growth. **B** Wnt ligands bind to Fzd and, via Ror/Ryk/Ptk7 receptors, increase intracellular calcium levels. Calcium activates CamK II, PKC, and calcineurin, which regulate NF-AT. NF-AT translocates to the nucleus and activates Wnt target gene expression. The Wnt/ Ca^2+^ pathway also influences cytoskeleton remodeling and cell shape changes. *Fzd*, Frizzled; *Ror*, receptor tyrosine kinase-like orphan receptor; *Ryk*, related to receptor tyrosine kinase; *Ptk7*, protein tyrosine kinase 7; *Dvl*, Dishevelled; *Daam1*, Dishevelled-associated activator of morphogenesis 1; *Rho/Rac*, Ras homolog/Ras-related C3 botulinum toxin substrate; *Rock*, Rho-associated protein kinase; *Jnk*, c-Jun N-terminal kinase; *CamK II*, calcium/calmodulin-dependent protein kinase II; *PKC*, protein kinase C; *CaN*, calcineurin; *NF-AT*, nuclear factor of activated T cells

The Pcp pathway mainly modulates cell polarity and motility. In this pathway, Wnts can potentially bind to the Fzd receptor. However, instead of Lrp5/6, which is the main co-receptor in the canonical pathway, the non-canonical pathway involves co-receptors such as receptor tyrosine kinase (Ryk), protein tyrosine kinase 7 (Ptk7), or receptor tyrosine kinase-like orphan receptor (Ror). The binding of this molecule triggers the activation of small GTPase proteins, such as Rho and Rac, via a Dvl-mediated process. This activation subsequently leads to the activation of Jnk and Rock signaling pathways, resulting in the remodeling of the cytoskeleton and the modulation of cell polarity. Additionally, this process also influences cellular development and survival (Fig. 2A) [12, 18, 19]. In the Ca^2+^-dependent pathway, Wnts augment intracellular Ca^2+^ levels, hence initiating the activation of Ca^2+^-dependent signaling pathways. It is related to cellular adhesion and migration, cell specialization, dorsolateral asymmetry, and restructuring of the cytoskeleton (Fig. 2B) [12, 13, 18, 19, 21].

### Wnt Regulators

Signaling pathways are controlled by both internal and external inhibitors and activators. Secreted frizzled-related proteins (SFRPs) and Wnt inhibitory factor-1 (Wif1) are examples of antagonistic effectors in this pathway. They bind to Wnt ligands or receptors and disrupt receptor activation. Since Wnt ligands and Fzd receptors share characteristics in both the canonical and non-canonical pathways, these inhibitors are effective in both pathways [5, 22, 23]. Two additional extracellular antagonists, Dickkopf (Dkk) and the Wise Sclerostin/Sost families, bind to Lrp5/6 and suppress the cascade. Since Lrp5/6 does not participate in the non-canonical cascade, these two antagonists only impact the canonical pathway [23, 24].

Within the Dkk family, Dkk-1 can attach to Lrp5/6 and demonstrates exclusive inhibitory properties. However, the impact of Dkk-2 may vary depending on the situation. Wise proteins are additional regulators of Wnt signaling. Their effect on the cascade can either stimulate or inhibit, depending on the circumstances. Reports have illustrated that Wise proteins can inhibit the activity of Wnt1 and Wnt3a and compete with Wnt8a in binding to Lrp6. Igfbp-4 is an insulin-like growth factor binding protein that can antagonize the canonical pathway by interacting with LRP6 and Fzd8 and competing with Wnt3a [25].

The R-spondins family (Rspo) serves as activators of the canonical pathway. Unlike the regulators outlined earlier, they function as transmembrane effectors rather than being secreted [25, 26]. To function synergistically, they require Wnts. Lrg4/5 serves as Rspo receptors, playing an essential role in the activity of Rspo. ZnRF3 and RNF43 are transmembrane E3 ubiquitin ligases that primarily inhibit the Wnt pathway. Rspo interacts with ZnRf3 via a process mediated by Lrg-4, which prevents the ZnRf3-mediated downregulation of Fzd and Lrp6. Rspo can induce Jnk signaling via the Pcp pathway by interaction with Sdc4 as well [25]. There are further positive regulators for the Wnt pathway. Norrin functions as a stimulator for the canonical pathway. It has been reported that it binds to Fzd4 and stimulates the pathway via Lrp5/6 [25]. Furthermore, Nogging, an antagonist of the Bmp pathway, can stimulate the non-canonical route [27].

The canonical and non-canonical pathways can mutually influence one another. Non-canonical activation may suppress the canonical pathway [26]. Wnt5a which is a non-canonical ligand can facilitate the degradation of β-catenin through a process that does not rely on glycogen synthase kinase-3β (GSK-3β) [28]. Furthermore, it has been demonstrated that Ptk7, a transmembrane protein involved in the Pcp pathway, can inhibit the canonical pathway [29].

### Wnt in CNS Development

Wnt signaling is a highly conserved pathway across metazoans, playing a crucial role in cell fate determination and morphogenesis. Its influence is particularly significant during neural development. This pathway performs a vital part in establishing the anterior–posterior and dorsal–ventral axes during embryonic development, which is essential for the early formation of the CNS [30, 31]. Research has highlighted that a defective Wnt signaling pathway is associated with neural development defects [32]. In addition, during the postnatal period, which is the main focus of this section, Wnt plays a crucial role in preserving and rejuvenating the structure and function of the nervous system. The role of Wnt in maintaining the neural system can be classified into two distinct categories.

### Wnt in Neurogenesis

Neural stem cells (NSCs), which are essential for the regeneration and differentiation of neurons, are affected by the Wnt cascade during both embryonic development and adulthood [19, 33]. Wnt signaling plays a dual role in neurogenesis; it induces proliferation during embryonic development and promotes differentiation in adult hippocampal neurogenesis. Nevertheless, the role of Wnt signaling in neurogenesis is complex. It has an impact on both the proliferation and differentiation of NSCs. Therefore, it is crucial to regulate it precisely in order to preserve both fully mature neurons and neural progenitors. Suppressing the expression of β-catenin through genetic manipulation causes early cell differentiation, while excessive activation of this pathway leads to heightened cellular specialization, resulting in a decrease in neural progenitors. Specifically, the modification of each of the Wnt components is linked to distinct behaviors, such as cell proliferation or differentiation. For instance, experimental investigations have demonstrated that LRP6 knockdown mostly affects cell differentiation rather than differentiation. On the other hand, enhanced production of Wnt3a results in greater self-renewal of NSCs and also their differentiation in mice embryos [34].

The Wnt pathway regulates neurogenesis in the adult hippocampus as well. For instance, Wnt3 and Wnt7a are responsible for NSC differentiation, proliferation, and self-renewal. The diminished release of Wnt3 by astrocytes is associated with impaired neurogenesis and impaired differentiation of NSCs during aging and may cause clinical impacts [35, 36]. Given the importance of Wnt in neurogenesis, studies have revealed a correlation between SET binding protein 1 (SETBP1) and Wnt pathways leading to regulate neurogenesis in the forebrain. Moderate to severe cognitive impairments can be caused by structural abnormalities in the brain and decreased neurogenesis in humans if the Wnt/β-catenin signaling pathway and the SETBP1 are disrupted during cortical neurogenesis [37]. In addition, the development of dendrites and the integration of newly born neurons into the hippocampus are both aided by the activation of the Wnt/β-catenin pathway, which in turn promotes NSC proliferation and differentiation [38]. These data indicate that the Wnt pathway plays a crucial role in regulating neurogenesis in the adult hippocampus and forebrain, influencing NSC functions and potentially impacting cognitive outcomes in aging and neurological disorders.

### Wnt in Synapse Formation

Synaptic structure and function are essential for the optimal operation of the nervous system. The Wnt signaling pathway plays a complex role in synaptic structure and function within the nervous system, influencing axonal and dendritic growth, synapse formation, impulse transmission, and synaptic plasticity through both canonical and non-canonical pathways. The Wnt signaling pathway is associated with the growth and development of both axons and dendrites, as well as the formation and adaptability of synapses [39–41]. Regarding this matter, it should be noted that Wnt3a and Wnt7a as ligands for canonical pathways participate in axonal branching and growth, in addition to their role in neurogenesis [33, 42]. Wnts activate Fzd receptors via the Wnt/Pcp pathway, which subsequently triggers Jnk signaling and leads to the remodeling of the cytoskeleton. This cascade is essential for the development of dendrites and the transmission of electrical impulses [41]. Wnt5a, as a non-canonical ligand, affects axonal growth and branching via the Pcp pathway and regulates synaptic activity and dendrite morphogenesis by stimulating the Wnt/Ca^2+^ pathway [41, 43]. Moreover, Dvl is a key mediator of Wnt signaling, and studies have demonstrated its role in axonal formation by activating different mediators. Any changes in the regulation of Dvl lead to axonal alteration [44].

Besides the illustrated role of Wnt in the regulation of synapse formation and development, both canonical and non-canonical are shown to be involved in impulse transmission, excitability, and neural plasticity in the nervous system [40]. For instance, the memory-related synaptic plasticity known as long-term potentiation (LTP) relies on Wnts [45, 46]. LTP is regulated by Wnt5a, and sFRP can disrupt LTP by inhibiting the Wnt pathway [47, 48]. Furthermore, neuronal excitability and synaptic plasticity depend on maintaining a balance between glutamatergic and GABAergic pathways, with sFRP shown to disrupt glutamatergic transmission by inhibiting the Wnt pathway, thereby impairing impulse transmission. Moreover, Wnt3a and Wnt5a are involved in neural cell oscillation in the entorhinal-hippocampus circuit. Wn3a impairs neural oscillation through the canonical pathway, and Wnt5a enhances it via the non-canonical cascade [19, 49]. Microglia communicate directly with astrocytes through the release of Wnt signaling molecules. This communication stimulates astrocytes to remodel their processes, thereby facilitating microglia-mediated synapse removal [50]. Modulation of the neuronal Wnt5a/Ca^2+^ signaling pathway is adequate to replicate critical signaling events downstream of receptor activation in neurons. These events include the activation of various kinases, elevation of somatic and dendritic Ca^2+^ levels, and enhanced trafficking of NMDARs to synapses [51].

## Wnt and Neurodegenerative Diseases

**Fig. 3:**
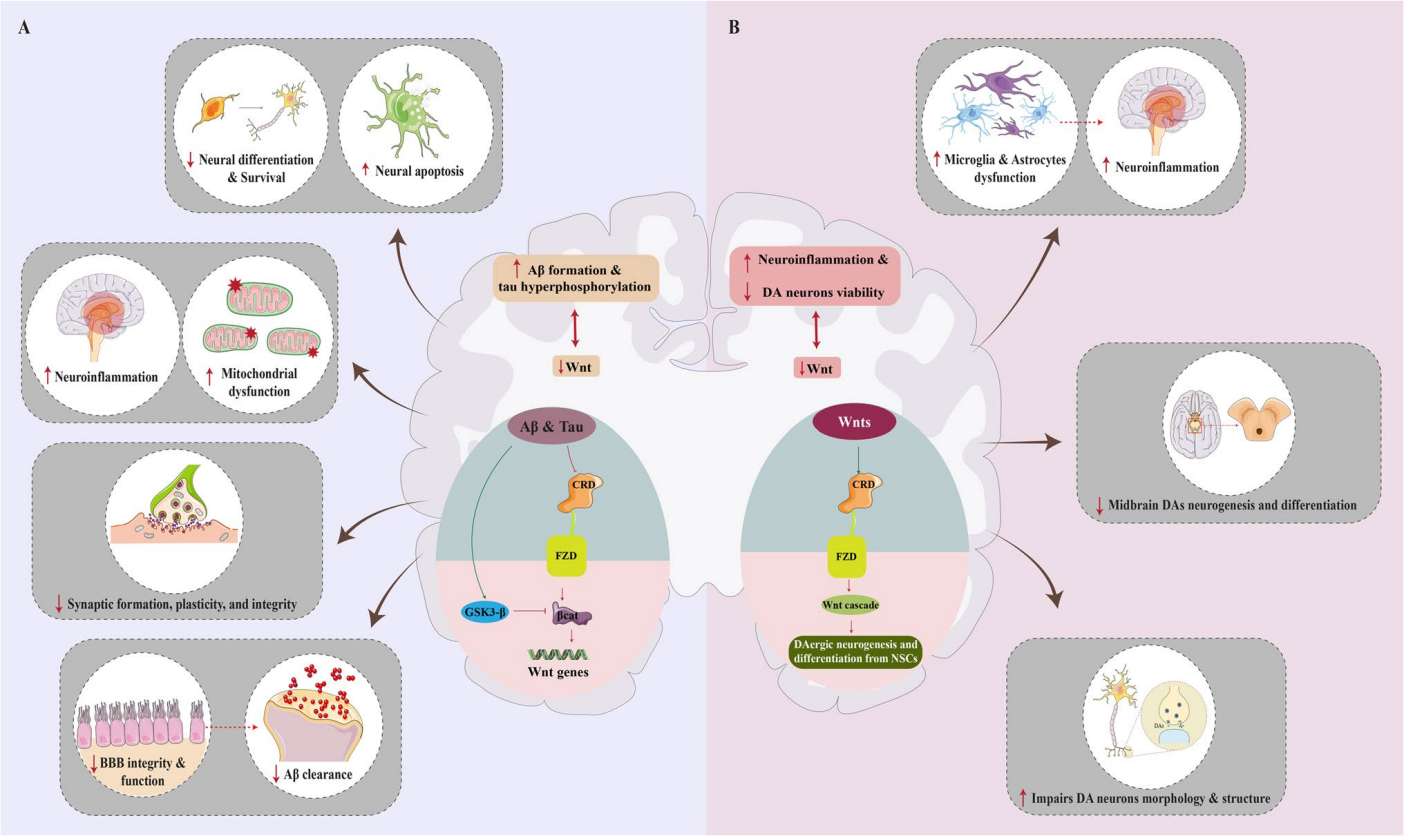
The role of Wnt signaling in the pathogenesis of Alzheimer’s disease (AD) and Parkinson’s disease (PD). **A** Amyloid-β (Aβ) can bind to the cysteine-rich domain (CRD) of the Frizzled (Fzd) receptor interfering with the binding of Wnts to their receptors that result in Wnt downregulation. Additionally, there is a bi-directional relation between the Wnt cascade and Aβ aggregation. A dysregulated Wnt signaling is associated with more Aβ accumulation. In addition to the interaction of Aβ with the Wnt receptors, it has been demonstrated that a high GSK3-β activity is correlated with Aβ accumulation as well. As a result, a disrupted Wnt signaling is associated with AD pathogenesis via several aspects. For instance, a suppressed Wnt cascade decreases neural viability and differentiation increases apoptosis, and disrupts synaptic formation and plasticity. Moreover, neuroinflammation and mitochondrial disruption which are responsible for AD pathogenesis contributed to a decreased Wnt activity. Eventually, in AD, Aβ clearance from the brain is reduced due to the altered blood–brain barrier (BBB) function which is a result of dysregulated Wnt signaling. **B** The role of Wnt in PD has been demonstrated via several mechanisms including increasing the neuroinflammation. An intact Wnt signaling is crucial for the regulation of inflammation by astrocytes and microglia in brain tissue. Furthermore, the differentiation of NSCs to dopaminergic (DA) neurons in the midbrain and DA neuron’s structure and activity are modulated by the Wnt cascade. Hence, in PD, dysregulated Wnt cascade results in a decrease in DA survival rate and differentiation

Wnt signaling has emerged as a significant area of study in the context of NDs, such as AD and PD. The analysis of crucial signaling pathways indicates the start and evolution of neurodegenerative diseases, with the early occurrence of synapse loss and neural death being a significant event and a crucial pathogenic factor associated with cognitive decline in patients. As previously discussed, Wnt signaling is essential for synaptic plasticity as it regulates the vesicle cycle, the movement of neurotransmitter receptors, neurotransmitter trafficking, and gene expression. Research indicates that insufficiency in the Wnt signaling pathway has a role in the development of neurodegenerative diseases like AD by causing the disintegration of the connections between nerve cells and the degradation of neurons [52]. Here, we explore common hypotheses regarding the pathogenesis of AD and PD, along with the role of Wnt signaling in the pathogenesis of each condition. Figure 3 illustrates a summary of the pathophysiological mechanisms associated with dysregulation of the Wnt signaling pathway in AD and PD.

### AD

#### Overview of the Pathogenesis

AD is a neurological condition marked by substantial deterioration of neurons and the occurrence of two specific protein buildups: amyloid plaques and intracellular neurofibrillary tangles (NFTs) [53]. Aβ cascade and p-tau have been identified as the main factors in this protein accumulation. The production of Aβ occurs when amyloid precursor protein (APP) undergoes breakdown in the membranes of neurons [54]. This process can result in the formation of AD due to either excessive synthesis or disruption in the clearance of Aβ. Soluble Aβ oligomers exert synaptotoxic effects at doses within the nanomolar range [55]. Microtubule assembly in neurons requires the microtubule-associated protein tau. The altered distribution of tau protein at the synapse, influenced by phosphorylation, can adversely affect neuronal function and potentially contribute to the pathogenesis of AD [56]. Tau pathology and the creation of NFTs are caused by Aβ formation and accumulation, which aids in the release and buildup of tau in neural plaques. The interaction between Aβ and tau, mediated by intermediates such as kinases, GSK-3β, CDK-5, and ERK, influences the immunological response, brain cell function, and signal transduction between neurons [57].

Furthermore, mitochondrial dysfunction can result in the accumulation of Aβ, the development of NFTs, and neurodegeneration. Aβ induces the expansion of axons and dendrites, disrupts signal propagation along axons in the hippocampus, and has a role in alterations of mitochondrial distribution and function. An increase in reactive oxygen species (ROS) due to excessive mitochondrial fission causes the impairment of large molecules and Aβ plaques to form [58, 59]. Increased ROS levels are detrimental because they disrupt cellular processes, generate pro-apoptotic proteins, and cause neuronal death in the CNS. Moreover, Aβ causes neurons to produce an overabundance of intracellular Ca^2+^ and an excessive influx of Ca^2+^, which in turn causes mitochondrial dysfunction, poor synaptic transmission and plasticity, and oxidative stress. These mechanisms may contribute to some of the cognitive decline associated with aging [60]. Neuroinflammation, which is a key component in the progression of NDs like AD, is triggered by the buildup of Aβ. Microglia perform a dual purpose, one of which is to regulate neuroinflammation and the other is to contribute to the development of AD. To avoid the formation of Aβ peptides and the production of pro-inflammatory chemicals that lead to neuroinflammation and the spread of Aβ and tau disease, the brain’s resident immune cells break them down. Recent investigations have confirmed the regulatory impact of Wnt signaling on inflammation associated with microglia. Modulation of Wnt signaling pathways has shown potential benefits for AD [61]. Gaining knowledge about microglia, Wnt pathways, and their interconnections may provide novel perspectives on the mechanisms behind neuroinflammatory processes in AD [62, 63].

#### The Role of Wnt in AD

Aβ accumulation in the course of AD which can cause neural death and synaptic malformation is tightly associated with Wnt pathway dysregulation through several mechanisms. In AD, it is believed that the activation of Wnt/β-catenin contributes to the neuroprotective effects on an adult’s brain. Therefore, its stimulation improves neural survival and differentiation and also decreases Aβ accumulation and Tau hyperphosphorylation. Nevertheless, there is a mutual relationship between Aβ and the Wnt dysregulation [64]. Studies revealed that the level of β-catenin in AD pathogenesis is decreased through different mechanisms associated with Aβ aggregation. The Aβ molecules bind to the CRD of the Fzd receptor interfering with the Wnt/β-catenin cascade activation via its ligands. Moreover, they can interact with GSK-3β, leading to its activation and the subsequent degradation of β-catenin. This process ultimately reduces the number of synapses and postsynaptic density since Wnt signaling is pivotal for synaptic formation [65]. On the other hand, Wnt/ β-catenin suppression causes more Aβ formation [64, 66]. Therefore, the elevated activity of GSK3-β is correlated with increased Aβ formation, which contributes to neural death and synaptic defects.

Clinical investigations indicate that the Wnt signaling pathway may contribute to cortical atrophy in patients with AD, with the right calcarine cortex being the brain region most affected by Wnt target genes [67]. Moreover, Aβ toxicity induces neuroinflammation and mitochondrial dysfunction, which are associated with changes in the Wnt cascade. In AD, mitochondrial abnormalities produced by Aβ-related pathology can be alleviated through modulation of both the canonical and non-canonical Wnt pathways. One example is the impact of the canonical ligand Wnt3a on mitochondrial membrane permeability. Conversely, the non-canonical ligand Wnt5a acts to inhibit and regulate the process of Aβ-induced mitochondrial fission [68–70]. It is important to note that these two pathways appear to exhibit different behaviors. The canonical pathway preserves and improves the arrangement and stability of synapses, while the non-canonical pathway triggers the retraction of synapses [39, 71]. In addition to the contrasting role of the non-canonical pathway in synaptogenesis, Wnt5a (a non-canonical ligand) is also involved in synaptic changes and cellular growth [72, 73].

Another significant mechanism established in AD pathogenesis is the compromised ability of the blood–brain barrier (BBB) to efficiently clear Aβ from the brain. The clearance of Aβ through the BBB is crucial for recovering AD pathologies. It has been demonstrated that the canonical pathway is disrupted in the BBB of various AD models, leading to impaired clearance of Aβ [74]. More interestingly, it has been revealed that impaired BBB function and brain endothelial cell (BEC)-related pathologies occur in the early stages of AD. Undoubtedly, Aβ diminishes the functionality of Wnt/β-catenin signaling in the BBB cells, resulting in increased leakage of blood components into the brain and reduced production of proteins that maintain the integrity and function of the BBB [75]. These findings underscore the potential of Wnt restoration to improve BBB function in the early stages of AD.

In addition, the activation of the Wnt/β-catenin pathway can hinder the formation of Aβ and decrease the levels of Aβ, which eventually prevents apoptosis and Aβ-induced synaptic impairment in neurons [76]. Given the neuroprotective impact of Wnt stimulation, it is reasonable to assert that it can restore and enhance synaptic plasticity and cognitive performance [52]. Furthermore, as mentioned above, there is interplay between the canonical and non-canonical pathways in synaptoxicity. Aβ aggregation induces synaptic impairments in a Dkk-1-associated manner. Dkk-1 downregulates the canonical and upregulates the Pcp pathway which finally alters dendritic expansion and growth and synaptic stability. These synaptic abnormalities are observed in the early stages of the disease prior to the onset of clinical manifestations [39, 71]. Therefore, re-establishing Wnt signaling could be a possible approach to treat AD since it could prevent synaptic loss, decrease Aβ synthesis, block tau phosphorylation, and control neuroinflammation [64].

### PD

#### Overview of the Pathogenesis

Another common neurodegenerative disease is PD. Neuroinflammation plays a major role in age-related neurodegenerative diseases like PD. Genetics, aging, infections, and the environment can all contribute to inflammation and changes in the substantia nigra pars compacta region, which can ultimately result in neuro-dopaminergic cell death [77, 78]. Autopsy examinations indicate the existence of activated microglia, T-cells, and immunoglobulin accumulation in the brain tissue of persons diagnosed with PD. T-cell activation can be induced by misfolded peptides derived from alfa-synuclein (α-SYN), a crucial protein in the pathophysiology of PD. The abnormal form of α-SYN aggregates in the form of oligomers or fibrils. Studies demonstrated that α-SYN aggregates can affect the creation of synaptic vesicles, the functioning of organelles, the metabolism of dopamine, and chaperone activity [79, 80]. Increased concentrations of α-SYN result in disruptions in the release of neurotransmitters, the provision of energy, and the functioning of mitochondria, which ultimately leads to neuroinflammation and the demise of neurons. PD is associated with an elevated quantity and enhanced activity of HLA-DR + microglia in the nigrostriatal area, which occurs throughout the process of neurodegeneration. This connection establishes a relationship between the immune response and neurodegeneration in PD. Pathological circumstances, such as the accumulation of α-syn, exposure to environmental toxins, or oxidative stress, activate the surface Toll-like receptors (TLRs) of microglial cells. Microglia become activated when α-syn proteins connect to TLRs in a clustered manner. Upon the activation of TLRs and their subsequent signaling pathways, the nuclear factor kappa B (NF-kB) is activated [81, 82]. This activation then stimulates the formation of the inflammasome and leads to an increase in inflammatory cytokines, including interleukin 1β (IL-1β), TNF, IL-6, TGFβ, nitric oxide species, ROS, and pro-apoptotic proteins. These effects are observed in the substantia nigra pars compacta, striatum, and cerebrospinal fluid. The inflammatory reactions result in damage and death of dopaminergic cells [77].

#### The Role of Wnt in PD

The Wnt/β-catenin pathway activity in dopaminergic neurons in an intact adult brain is regulated by microglia and astrocytes. This regulation is crucial for maintaining the normal structure, function, and renewal of midbrain dopaminergic neurons (mDA) [83]. Moreover, the astrocytes and microglia are shown to regulate inflammation which is associated with Wnt dysregulation [84]. Furthermore, the role of multiple Wnt ligands and Wnt inhibitors in DA neuron’s morphogenesis, proliferation, differentiation, and function has been illustrated. Both the canonical and non-canonical participate in DA neuron morphogenesis, proliferation, differentiation, and function. For instance, Wnt1, Wnt2, Wnt3a, and Wnt5a are studied earlier. Dkk-1 and sFrp 1/2 are also mentioned in studies in neurogenesis and dopaminergic activity of neurons as well [85]. This signaling pathway has a substantial impact on the onset and progression of PD. Studies indicate that the disruption of the Wnt/β-catenin pathway is linked to the development of the pathogenesis of PD and that it can control a comprehensive process for the preservation and restoration of dopaminergic neurons in PD, which is considered a potential therapeutic target for PD, as it regulates various cellular functions and has neuroprotective and regenerative capacities [85–87].

L’Episcopo et al. undertook a study to evaluate the impact of acute 1-methyl-4-phenyl-1,2,3,6-tetrahydropyridine (MPTP) exposure on the decline and restoration of nigrostriatal dopaminergic functioning, which is controlled by Wnt1 (a crucial transcript produced from astrocytes). The researchers discovered that Wnt1 stimulated the formation of new neurons and DAergic neurogenesis from adult stem/neuro progenitor cells in the midbrain. They also observed that inhibiting the Wnt/Fzd signaling pathway using Dickkopf-1 reversed the protective effects of astrocytes against MPTP toxicity. Furthermore, they demonstrated that the lack of Wnt1 transcription in middle-aged mice and the recovery of DAergic neurons may be reversed through the pharmacological activation of Wnt/β-catenin signaling. The Wnt/β-catenin pathway is considered a potential therapeutic target for PD, as it regulates various cellular functions and has neuroprotective and regenerative capacities [87].

## Wnt-Targeted Therapeutic Interventions

**Fig. 4:**
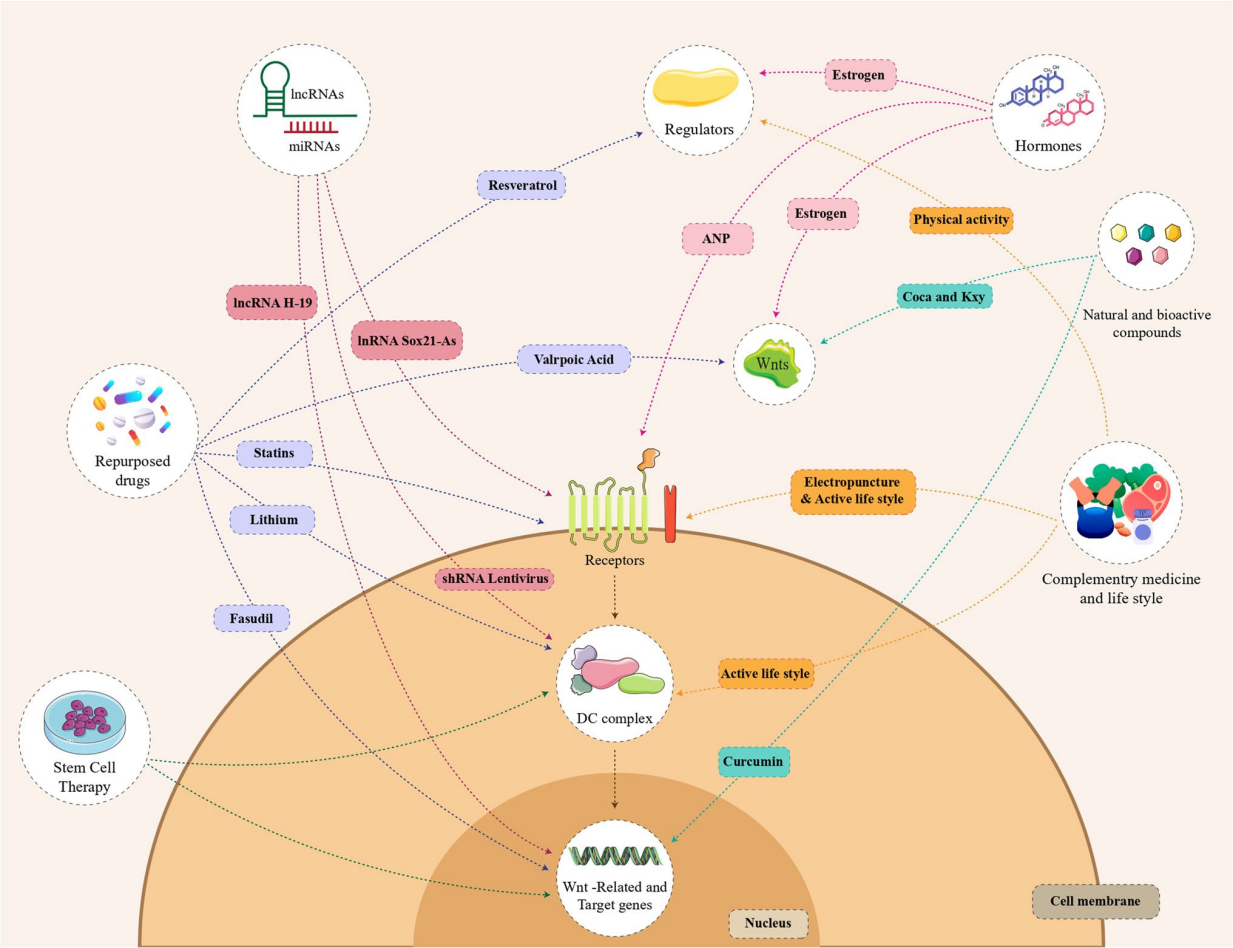
As shown in the figure, there are several therapeutic methods for neurodegenerative diseases that work by affecting the pathways involved in Wnt signaling. Among these treatments, lncRNAs and miRNAs, repurposed drugs, stem cell therapy, complementary medicine, hormones, and the use of natural and bioactive compounds can be mentioned. In the figure, some examples of these treatment methods and the signaling path of their effect points are drawn

Given the comprehensive data discussed earlier in this study, it is not out of the question to highlight the potential of Wnt modulation in the treatment of NDs. Here, we explored studies highlighting the Wnt pathway as a novel therapeutic approach for addressing the treatment of AD and PD, exploring the roles of individual components within the Wnt signaling pathway. Figure 4 illustrates an overview of Wnt-targeted intervention in the treatment of ND.

### AD

Numerous studies have focused on various components and mediators of the Wnt pathway in order to ascertain their precise significance in the pathogenesis of AD and to restore the normal functioning of Wnt in AD as a treatment strategy [88]. We addressed the alteration of each component ranging from receptors to genes and discussed their potential as a treatment for AD (Table 1).

**Table 1:** A summary of recent studies on Wnt signaling components for AD treatment

Agent/intervention	Level of manipulation	Targeted component	In vivo/in vitro	Outcome of the study	Year	Reference
Kxs	Multiple levels	Wnt1, β-catenin, Gsk-3β,	In vivo	Mechanism identification, Wnt/β-catenin restoration, improve neural damage and cognitive impairment	2023	[89]
Cyanidin	Multiple levels	Wnt3a, Wifs, β-catenin	In vitro	Wnt/β-catenin restoration, promote nerve growth and differentiation, alleviate tau-hyperphosphorylation	2023	[90]
Electroacupuncture	Multiple levels	Wnt5a, Dvl 2, Camk 2, Fzd 2	In vivo	Wnt/β-catenin restoration, decrease Aβ formation, enhance neurogenesis	2023	[91]
Cocoa	Ligand	Wnt3a	In vivo	Wnt restoration	2023	[92]
Sdkd	Ligand	Wnt3a	In vivo in vitro	Mechanism identification, reduce neural loss, enhance NSCs proliferation	2023	[93]
Wnt2b overexpression	Ligand	Wnt2b	In vivo in vitro	Improve mitochondrial dysfunction, alleviate neural damage	2023	[94]
Las	Receptor	Lrp6	In silico	Wnt restoration	2023	[95]
Bacopa monnieri	Dc components	Gsk-3β	In vivo	Regulate mitochondrial dysfunction, Wntβ/-catenin restoration	2023	[96]
AD201 suppression	Regulator	sFrp	In vitro	Mechanism identification, Wnt/β-catenin restoration	2023	[97]
5-methoxyindirubin-3'-oxime	Regulator	CXXC5	In vivo	Mechanism identification, Wnt/β-catenin restoration, decrease Aβ and improve cognitive function	2023	[98]
Mor, Tml, Tmq	Multiple levels	Wnt3a, Gsk-3β	In vivo	Wnt/β-catenin restoration, decrease Aβ formation, inflammation and apoptosis	2022	[99]
Neural stem cell secretome	Multiple levels	Gsk-3β, Wnt related genes^a^	In vivo	Modulating Wnt/ catenin	2022	[100]
OGDHL overexpression	Ligand	Wnt7a	In vivo in vitro	Decrease neuroinflammation, Aβ plaque and tau phosphorylation	2022	[101]
Fusadil ^b^	Ligand	Wnt4	In vivo	Wnt restoration, mechanism identification	2022	[102]
OptoLrp-6	Receptor	Lrp-6	In vitro	Wnt/β-catenin restoration, improve Aβ associated BBB damage	2022	[75]
DNLA	Dc components	Gsk-3β	In vivo in vitro	Improve and preserve synapses integrity, mitochondrial dysfunction and suppress Aβ production	2022	[65]
La	Dc components	Gsk-3β	In vitro	Decrease apoptosis, and β-catenin degradation	2022	[103]
MFSs	Dc** component	Gsk-3β	In vivo	Mechanism identification, enhance cognitive function and improve neurons survival	2022	[104]
Ginsenoside Rg1 ^c^	Dc component	Gsk-3β, β-catenin	In vivo	Decrease apoptosis, oxidative stress and neuroinflammation	2022	[105]
Fasudil	Intracellular cascade component	Rock	In vivo in vitro	Wnt/PCP downregulation	2022	[106, 107]
Simvastatin	Regulator	Dkk-1	In vivo	Wnt/β-catenin restoration Normalized defective dendrites,	2022	[108]
LME	Multiple levels	Gsk-3β, Dvl, β-catenin	In vivo	Increase neurogenesis and improve cognitive Impairment,	2021	[109]
Tadalafil ^d^	Multiple levels	Gsk-3β, cyclin D1	In vivo	Preservation of neural integrity and enhance cognitive behavior	2021	[110]
Bergapten ^d^	Multiple levels	Gsk-3β, Cyclin D1	In vivo	Preservation of neural integrity and enhance cognitive behavior	2021	[110]
Class α-glucosidase inhibitors and PPARs agonist	Receptor	Lrp5/6	In silico	Wntβ/-catenin restoration	2021	[111]
Valproic acid	Multiple levels	Wnt3a, Gsk-3β	In vivo	Increase neurogenesis and improve behavioral and cognitive analysis	2019	[112]
lncRNA SOX21-AS1	Receptor	FZD3/5	In vivo	Decrease neural apoptosis and oxidative stress, Wnt restoration	2019	[113]
Fluoxetine	Dc components	Gsk-3β	In vivo	Decrease Aβ formation and apoptosis and preserving synapses	2018	[114]
Gallocyanin	Regulator	Dkk-1	In vitro	Wntβ/-catenin restoration	2018	[115]
Sodium selenate	Dc components	Gsk-3β	In vivo	Decrease Aβ formation	2017	[116]
Selenomethionine	Dc component	Gsk-3β	In vivo	Stimulate neurogenesis and NSCs differentiation	2017	[117]
Cholinesterase inhibitors	Dc component	Gsk-3β,	In vivo in vitro	Decrease Aβ formation, p-tau and oxidative stress, Wnt restoration	2016	[118]
Simvastatin	Receptor	Lrp-6	In vivo	Decrease Neural apoptosis	2016	[119]
Osthole	Dc component	Gsk-3β	In vitro	Decrease apoptosis, increase NSCs proliferation and differentiation	2015	[120]
Ethosuximide	Multiple levels	Gsk-3β, Dkk-1	In vivo	Increase NSC proliferation and differentiation	2015	[121]
Mesenchymal stem cell	β-catenin	β-catenin	In vitro	Stimulate neurogenesis and neural differentiation	2015	[122]
Physical activity	Multiple level	Dkk-1, Wnt3a, Lrp5/6		Wnt restoration	2014	[123]
Wasp-1	Ligand	Wnt3a	In vivo	Improve cognitive function and synaptic impairment	2014	[124]
Foxy-5	Ligand	Wnt5a	In vivo	Improve cognitive function and synaptic impairment	2014	[124]
Curcumin	Multiple levels	Gsk-3β, β-catenin, CyclinD1	In vitro	Wnt/β-catenin restoration	2011	[125]
E2 ^e^	Regulator	Dkk-1, wnt3a	In vivo	Neuroprotection	2008	[126]

Abbreviations: *Kxs*, Kai-Xin-San; a classic product clinically effective for amnesia and AD cognitive impairment

*Sdkd*, Sanwei DouKou decoction; a classic Chinese prescription for AD. *Las*, LAS 29757582, LAS 29984441, and LAS 29757942; these three compounds interfere with Dkk-1 and Lrp6 binding. *Mor*, Morin; *Tml*, Thymol; *Tmq*, Thymoquinone; *OGDHL*, oxoglutarate dehydrogenase-like; *DNLA*, dendrobium nobile Lindl. Alkaloids; *La*, alpha-lipoic acid; *MFSs*, miracle fruit seeds.; *LME*, lycopene-loaded microemulsion; *MFS*, miracle fruit seed; *PPARs*, peroxisome proliferator-activated receptors; Sos21-As1, SRY-box transcription factor 21 antisense divergent transcript 1; *E2*, 17β-Estradiol

^a^PI3K, ERK, MAPK, and Akt are several Wnt-related genes

^b^The efficacy of Fusadil in improving the condition of AD models has been demonstrated. Nevertheless, the therapeutic benefits are not only attributed to the restoration of Wnt

^c^This study’s findings provide evidence for the concept of the neuroprotective impact of Wnt restoration. However, to be more specific, Ginsenoside Rg1 decreases the expression of β-catenin along with reducing Gsk-3β and Caspase 3 expression and also inhibits the phosphorylation of Gsk-3β and β-catenin. Furthermore, the expression of BACE1 was diminished following administration of Rg1. Ultimately; the overall outcome affirmed the promise of Rg1 for clinical studies. This work has unveiled the intricacy of signaling pathways and their intercommunication

^d^This study has indicated the protective effect of these drugs is derived from their ability to regulate the interaction between multiple signaling cascades, including the Wnt pathway

^e^According to this study, the protective impact of E2 (17beta-Estradiol) on Tau phosphorylation is accomplished via inhibiting Dkk-1 upstream (Jnk/Jun signaling) and increasing Wnt3a levels directly

#### Ligand Targeting

In the preceding sections, we explored the role of Wnt signaling and various Wnts in neurogenesis and synaptic development. Multiple studies have demonstrated a correlation between reduced levels of both the canonical and non-canonical Wnt ligands in the brain affected by AD, suggesting the potential therapeutic advantages of boosting Wnts levels to reinstate Wnt signaling for treating neurodegenerative conditions [89, 90, 94]. For instance, similar to Wnt1 which participates in neurogenesis, Wnt3a is proven to be associated with neurogenesis, axonal growth, and neural impulse activity, and studies indicate that the secretion of Wnt3a is modified in aging and AD. Similar to Wnt3a, Wnt7a and Wnt5a are involved in multiple aspects of neural development and function, and they also play roles in the pathogenesis of AD [127]. During AD, even in its early phases, pathologies associated with Wnt5a alteration are demonstrated. For instance, In AD mice, a significant decrease in the expression of Wnt5a within the hippocampus, coinciding with impaired neurogenesis in the dentate gyrus, has been observed [91].

Concerning the different Wnt ligands associated with AD pathogenesis, various approaches can be employed to stimulate the Wnt pathway (Table 1). One of the considerable interventions is the repositioning of FDA-approved medications. Drug repositioning is an innovative approach to address the challenges of developing new medications. While the extent of drug repositioning’s potential in influencing the Wnt pathway has not been thoroughly examined, several investigations have explored the regulatory effects of established pharmaceuticals on the Wnts and Wnt signaling components. For instance, valproic acid can enhance the Wnt3a stimulation, but its therapeutic impact is not specified to the Wnt3a. It has been found that valproic acid can improve cognitive and behavioral function and neurogenesis in a mouse model of AD [112]. Moreover, another drug, Fasudil, has been suggested for the treatment of AD. This medication can elevate levels of Wnt4a; however, its therapeutic benefits extend beyond mere Wnt restoration [106].

Natural compounds are increasingly investigated for their potential application in the treatment of AD. The modulatory effects of various herbal compounds, such as Kai-Xin-San and Cocoa, on Wnt signaling have been investigated for their potential to alleviate AD symptoms in both in vitro and in vivo studies. These experimental investigations have shown encouraging results, demonstrating the prevention of neuronal damage and enhancement of cognitive function following the administration of these herbal substances in the treatment of AD [89, 92]. In addition to pharmaceutical treatment, gene-based interventions have also shown promising results. For example, overexpression of oxoglutarate dehydrogenase-like has been demonstrated to reduce neuroinflammation, Aβ and plaque formation, and tau hyperphosphorylation through stimulation of Wnt7a [101]. Similarly, the upregulation of Wnt genes, such as Wnt2b, has been reported to have a positive impact on AD pathogenesis [94]. Prioritizing Wnt signaling has the potential to revolutionize AD therapy. While methods aimed at restoring the Wnt cascade through its ligands have shown promise, further clinical investigations are essential to address any unexpected outcomes. Table 1 shows additional insights into comparable therapies for AD treatment.

#### Receptor Targeting

Employing diverse strategies to target Wnt receptors and co-receptors may address therapeutic challenges in AD. LRP is downregulated in the brains of individuals with AD. Moreover, in transgenic animal models lacking LRP5/6, there were aberrant changes in synapse formation and increased deposition of Aβ, indicating a mutually beneficial relationship between the suppression of the canonical route and the aggregation of Aβ. In this context, the restoration and overexpression of LRP could potentially serve as a therapeutic approach for AD [128]. To achieve this goal, various strategies have been considered, including medication interventions and the innovative approach of drug repositioning. For example, the Wnt restoration and upregulation of LRP-6 following the application of simvastatin, a potent antihyperlipidemic medication, improves memory and granule cell maturation in a mouse model of AD [108]. By activating the Wnt/β‐catenin signaling pathway, simvastatin also inhibits neural cell apoptosis, reduces tissue damage, and improves functional recovery after acute neural tissue injury [119].

Furthermore, in silico studies can identify possible drug interactions between known pharmaceuticals and Wnt receptors. For example, antidiabetic drugs are known to stimulate the Wnt pathway via LRP5/6. Given LRP’s role in AD pathogenesis, this interaction has the potential to significantly impact AD treatment [111]. Additionally, studies have shown that modifying lifestyle has a positive effect on Wnt restoration in the setting of AD [123]. Besides, a correlation between Vitamin D and LRP-1 has been reported, suggesting its potential involvement in Aβ clearance in patients with AD [129]. Furthermore, in the previous section, we discussed the involvement of Wnt signaling in BBB dysfunction, which impairs Aβ clearance. Hence, recovering this pathway leads to enhanced Aβ clearance and improved clinical outcomes. In a study conducted by Wang et al., it was demonstrated that the activation of Wnt/β-catenin signaling through optogenetic LRP6 stimulation can effectively inhibit and reverse the Aβ-induced BBB damage in both in vitro and animal models of AD [75].

In addition to LRP, Fzd serves as the primary receptor in both the canonical and non-canonical pathways. The modification of its activity has been demonstrated to play a role in the progression of AD pathogenesis [65]. As mentioned, the stimulation of Fzd contributes to downstream cascade activation, which leads to neuroprotective effects. To underscore this phenomenon, experimental investigations have been conducted to examine various strategies, including the utilization of complementary medicine like electroacupuncture for neuroprotection in models of AD. These studies provided conclusive evidence through the stimulation of Fzd receptors [91, 113]. Ultimately, it is worth mentioning that targeting Fzd receptors could potentially be utilized for diagnosing abnormalities in the Wnt signaling pathway in the NDs [130].

#### Modulation of β-Catenin and Dc Components

The β-catenin Dc components play an important role in maintaining cellular homeostasis via the regulation of Wnt signaling pathway activity and controlling the levels of β-catenin. β-catenin can decrease the expression of β-amyloid precursor protein cleaving enzyme-1 (BACE1), which suggests the potential of Wnt/β-catenin regulation in the treatment and prevention of AD [76]. Moreover, some studies investigated the interventions that upregulated β-catenin to promote its-associated gene expression to evaluate the neuroprotective benefits of the interventions [90, 109]. Given these significant findings, therapeutic interventions that target and reduce the activity of the Dc complex may exert a potential positive effect on AD treatment.

GSK-3β serves as the main controller of the Wnt pathway and is a subunit of Dc. It participates in various interactions with other signaling pathways. Therefore, it has been and continues to be the main area of interest for many interventional and experimental investigations conducted on various disorders [131, 132].

In addition, Aβ triggered the activation of the Wnt/Pcp pathway in the development of AD. The enhanced Pcp pathway subsequently amplifies GSK-3β activity, leading to the suppression of the canonical pathway and the promotion of Tau-related impairments [106]. Hence, GSK-3β inhibition is effective in reducing Aβ production, improving synaptic degeneration induced by Aβ, and decreasing Aβ formation and neural apoptosis. Multiple agents including repurposed medications have been studied regarding their role in regulating the GSK-3β activity. For instance, lithium chloride, which mimics Wnt signaling by inhibiting GSK-3β, has been widely investigated. Studies have demonstrated that lithium chloride can disrupt Aβ pathogenesis and improve the deficit in spatial learning [133]. Therefore, it has been suggested that the inhibition of GSK-3β or the reduction in its gene expression can be regarded as having similar outcomes [104, 121, 134, 135]. Moreover, numerous other medications, including fluoxetine, cholinesterase inhibitors, and ethosuximide, as well as compounds like curcumin, have been identified to negatively affect GSK-3β. This inhibitory effect results in decreased Aβ formation, reduced tau hyperphosphorylation, decreased neural apoptosis, and increased NSC proliferation [114, 118, 121, 125]. However, it has been reported that the application of some compounds, such as tadalafil and bergapten, improves cognitive function and histological architecture in a mouse model of sporadic AD. This improvement is associated with enhanced protein expression of GSK-3β, increased hippocampal levels of Wnt3a, and reduced expression of β-catenin [110].

#### Modulation of Wnt Regulators

Targeting the Wnt/β-catenin pathway has also shown significant therapeutic potential. Earlier, we discussed the role of Dkk-1 in AD [19]. Typically, the expression of Dkk-1 is low in a healthy intact brain, but an elevated level of Dkk-1 expression is linked to cell death. This phenomenon has also been demonstrated in investigations conducted on the brains of AD models. It has been demonstrated that Aβ induces the Wnt/Pcp pathway, leading to increased synthesis of Dkk-1 during AD [49]. Subsequently, the Dkk-1 has been illustrated to be involved in Aβ-induced Wnt/β-catenin suppression. Furthermore, the presence of a significant amount of Dkk-1 expression caused by the accumulation of Aβ is linked to an elevation in tau-hyperphosphorylation [106]. Aβ accumulation leads to synaptoxicity in a Dkk-1-dependent process as well. Marzo et al. have investigated the role of Dkk-1 in synaptogenesis in AD mouse models and revealed that Dkk-1 significantly impairs synaptic function via GSK-3β and Rho/Rack signaling inhibition [136]. Therefore, targeted intervention to inhibit Dkk-1 can be considered reasonable to recover the altered Wnt pathway. Studies have illustrated this issue with different influential compounds. It has been demonstrated that engaging in physical activity and the presence of estrogen can decrease the level of Dkk-1 and upregulate the Wnt pathway [126, 137]. Moreover, statins have been proven to have suppressive effects on Dkk-1 [138]. Furthermore, gallocyanine dyes, potent Dkk-1 inhibitors, interact with LRP and Dkk-1, regulate the Wnt pathway, and inhibit prostaglandin J2-induced tau phosphorylation at serine 396 [139]. Given the critical role of tau hyperphosphorylation in AD and the significant impact of inflammation on the pathogenesis and progression of NDs, this intervention holds promise for effectively treating AD.

sFrp, like any other Wnt antagonist, can affect the pathway in AD pathogenesis. The elevated levels of sFrp are related to Wnt suppression which has been shown in the AD experimental model [97]. Hence, therapeutic approaches focused on the sFrp can be promising. The therapeutic role of targeting sFrp in other disorders, like cancer, is proven [22]. There is compelling evidence supporting the use of sFrp as a therapy for NDs. AD201, which is a member of the sFrp family, has been proven to be associated with AD manifestation in both in vitro and in vivo studies. It has been demonstrated that elevated levels of AD201 contribute to Wnt suppression, synaptic loss, ROS-related neurodegeneration, and cognitive decline [97]. Therefore, targeting this component with RNAi-based inhibition techniques would be a promising therapeutic approach, with potential benefits including improved neurodegeneration, enhanced mitochondrial activity, and reduced oxidative stress damage.

In addition to sFrp, the CXXC5 which is another negative regulator of the canonical pathway has been demonstrated to be increased during AD. This finding has been investigated in human hippocampal tissues and AD animal models and reported to be associated with a drop in the level of β-Catenin. Hence, it was initially hypothesized that the suppression of CXXC5 via 5-methoxyindirubin-3'-oxime can reinstate the Wnt activity and improve the outcome of AD [98]. Moreover, therapeutic interventions targeting Wifs along with other Wnt components could modulate tau-hyperphosphorylation by restoring the Wnt/β-catenin signaling cascade. Cyanidin has been studied for this purpose and has been shown to significantly enhance the expression of Wnt3a mRNA while reducing Wif1 expression [90].

Agonists of the Wnt pathway represent another potential target for therapeutic strategies. The Wnt signaling pathway can also be stimulated via its enhancers. For instance, sirtunin-1 (Sirt-1) is a positive regulator of the Wnt pathway. Studies have revealed the therapeutic importance of resveratrol in NDs, like AD. Resveratrol, a potent Sirt-1 enhancer, has the potential to enhance neurogenesis and improve memory loss through the Wnt signaling pathway in AD [140]. Furthermore, riluzole, a glutamate modulator, may exert beneficial effects in AD by targeting the canonical WNT/β-catenin pathway via CTNNB1-associated mechanism to modulate the glutamatergic pathway, reduce oxidative stress, and mitigate neuroinflammation [141, 142].

### PD

The involvement of Wnt signaling in PD pathogenesis has been supported by evidence from both in vivo and in vitro studies [85]. Restoring Wnt/β-catenin is believed to have a neuroprotective effect on PD [143]. Ranging from ligands to related genes, there are multiple therapeutic targets in the Wnt pathway to achieve neuroprotection in PD. Multiple medications and chemical compounds have been suggested to exhibit neuroprotective effects in PD via restoring Wnt, including vinpocetine, ginsenoside Rg1, fasudil, and D1 dopamine receptor agonist [134, 144–147]. Among these potential treatment options, some include repurposed medications, such as the oral hypoglycemic agent empagliflozin, which are discussed below (Table 2) [148].

**Table 2:** A synopsis of Wnt signaling manipulation for Parkinson’s disease

Agent	Level of manipulation	Targeted component	In vivo/in vitro	Study outcome	Year	Reference
Empagliflozin	Multiple levels	Wnt3a, β-catenin	In vivo	Wnt/β-catenin restoration	2023	[148]
Vinpocetine	Multiple levels	Wnt1, β-catenin, C-myc	In vivo	Wnt/β-catenin restoration, alleviate Os and dyskinesia and downregulate α-synuclein expression	2023	[144]
Mangiferin	Multiple levels	Wnt1, Gsk-3β	In vitro and in vivo	Wnt/β-catenin restoration Decreasing OS, enhance mitochondrial membrane potential	2023	[149]
CREB1	Dc component	Axin-2	In vitro and in vivo	Wnt/β-catenin restoration, decrease inflammation, apoptosis	2023	[150]
L-theanine	Multiple levels	Wnt3a, Wnt5a, β-catenin, Tcf1/7, lef7	In vivo and in vitro	Wnt restoration, alleviate inflammation	2022	[151]
Roucongrong	-	-	In vivo and in vitro	Wnt/β-catenin restoration, increase neural survival	2021	[152]
lncRNA H19	β-catenin	β-catenin	In vivo	Wnt/β-catenin restoration	2020	[153]
Tormentic acid	Multiple levels	β-catenin, Gsk-3β	In vivo	Wnt/β-catenin restoration, decrease apoptosis and increase neural viability	2020	[145]
HIF-1α /miR-128-3p	Dc components	Axin1	In vivo	Wnt/β-catenin restoration, Axin1 down regulation, Alleviate neural apoptosis	2020	[154]
Mulberrin	Multiple levels	Wnt3a, β-catenin, C-myc	In vivo and in vitro	Wnt/β-catenin restoration, alleviate neuroinflammation and Os	2019	[155]
D1 receptors agonists	Multiple levels	Wnt3a, β-catenin, Axin-2, Lef1, CyclinD1,	In vivo	Wnt/β-catenin restoration, increase neurogenesis, alleviate non-motor manifestation	2019	[146]
ANP	Receptor	Fzd1/2	In vitro	Wnt/β-catenin restoration	2018	[143]
Axin2- shRNA lentiviruses	Dc component	Axin-2	In vivo	Wnt/β-catenin restoration, alleviate apoptosis, Os and mitochondrial dysfunction	2018	[156]
Ginsenoside Rg1	Multiple levels	Wnt1a, Gsk-3β, β-catenin	In vivo and in vitro	Wnt restoration, alleviate neuro immobility, neuroprotection	2015	[134]
Fusadil	Multiple levels	Wnt1a, Fzd1, β-catenin	In vivo	Wnt/β-catenin restoration, improved motor activity and decrease inflammation	2015	[147]
XAV-nano-stroma delivery	Dx component	Axin 1/2	In vivo and in vitro	Enhance DA neuron survival	2014	[157]
AR-AO14418	Dc component	Gsk-3β	In vivo	Wnt/β-catenin restoration, neurogenesis, alleviate motor symptoms	2014	[158]^a^

Abbreviations: *ANP*, atrial-natriuretic peptide; *HIF-1α*, hypoxia inducible factor-1α; *miR-128-3p*, microRNA-128-3p

^a^Some of the studies mentioned in Table 2 were not specifically and directly conducted to examine the effect of specific agents in Wnt modulation

#### Ligand Targeting

Multiple Wnt ligand of both canonical and non-canonical pathways is associated with the regulation of pathologically altered processes in PD pathogenesis [85]. The researchers discovered that Wnt1 stimulated the formation of new neurons and dopaminergic (DA) neurogenesis and differentiation from adult stem/neuro progenitor cells in the midbrain [87]. Moreover, there is interplay between Wnt1 and Wnt5a contributed to DA development. Investigations suggest that Wnt1 facilitates the proliferation of DA neurons, whereas Wnt5a, known for its involvement in neural morphogenesis, has been shown to inhibit proliferation. However, Wnt1 is necessary for the activation of the Wnt5a-induced Pcp pathway that regulates the morphogenesis of DA neurons [85]. Wnt2 and Wnt3a have also been demonstrated to have important roles in neurogenesis and differentiation. As a result, targeting Wnts can be considered a novel strategy for the treatment of PD. In pursuit of this objective, medicines and other substances have been thoroughly examined, yielding encouraging outcomes. Moreover, L-theanine is another chemical that has shown promise in treating PD. In PD mouse models, administration of L-theanine improves motor dysfunction by stimulating and restoring the Wnt pathway. This modulation subsequently changes the levels of inflammatory cytokines, such as IL-6 and TNF-α, and pro-apoptotic proteins, like P53. Due to the targeting of the Wnt components, including Wnt3a, Wnt5a, the Dc components, and Wnt-related genes (such as Tcf and Lef), L-theanine is implicated as a neuroprotective drug with potent anti-inflammatory effects [151]. In addition, fasudil has been suggested to improve the outcome of PD. This Rho kinase inhibitor reduces the levels of inflammatory cytokines, such as IL-1β, and also increases the expression of multiple components of Wnt including the Wnt1. This restoration of Wnt signaling helps alleviate behavioral and motor dysfunction in mice model of PD [147].

#### Receptor Targeting

Targeting Fzd, the upstream receptor of the Wnt pathway to upregulate this pathway, has been shown to exhibit therapeutic effects in different disorders. Here, in PD, Fzd has been targeted to achieve neuroprotective effects as well [143]. For instance, the extracellular region of Atrial Natriuretic Peptide (ANP) encompasses two Fzd-like cysteine-rich domains, Fzd1 and Fzd2, which act as receptors for Wnt signaling [159]. Activation of the Wnt pathway by ANP exerts a neuroprotective effect, particularly when these cellular systems are exposed to neurotoxic insults such as 6-OHDA, which mimics the neurodegeneration observed in PD [143]. Furthermore, the administration of fasudil resulted in elevated expression levels of Fzd1 as well as Wnt1 and β-catenin in a mouse model based on MPTP-induced PD. This elevation correlated with an increased number of tyrosine hydroxylase neurons and a significant enhancement in motor performance [147].

#### Modulation of β-Catenin and Dc Components

The activity of the Wnt/β-catenin pathway relies on β-catenin [9]. Upregulation of β-catenin, either alone or in combination with modifications to other elements of the Wnt signaling pathway, is advantageous in promoting neuroprotection. Multiple studies have employed drugs and interventions to increase the activity of the Wnt/β-catenin signaling pathway by enhancing the expression of the β-catenin gene. Long non-coding RNAs (lncRNAs) and microRNAs (miRNAs) have been shown to offer neuroprotection through distinct mechanisms. It has been indicated that increased levels of lncRNAs H19 can prevent DA neuron loss in PD animal models via Wnt activation [153]. Moreover, miR-23b-3p-enriched mesenchymal stem cell exosomes have been found to promote neuronal autophagy by increasing the expression of β-catenin and regulating the Wnt signaling pathway in both rat and cellular models of PD [160]. Furthermore, targeting the Dc components like Axin and Gsk-3β to restore the Wnt in PD experimental models of PD has been demonstrated to have protective effects. Downregulation of GSK-3β expression has also been demonstrated to effectively alleviate PD symptoms in experimental studies [134, 145]. Moreover, genetical knockdown of Axin via shRNA lentivirus, a negative regulator of Wnt/β-catenin signaling and a scaffold protein in Dc, improves behavioral functions, protects the nigral DAergic neurons, and promotes mitochondrial biogenesis in substantia nigra pars compacta in 6-OHDA-induced rat models of PD [156].

Previously discussed natural and bioactive compounds may have the potential to treat NDs by modulating Wnt signaling. Studies provide evidence for the therapeutic impact of mangiferin, a bioactive substance derived from mango, in PD. In PD experimental models, mangiferin has been shown to restore Wnt signaling, decrease oxidative stress, improve mitochondrial function, and alleviate motor impairments by inhibiting the expression of AKR1C3, a Wnt inhibitor [149]. Therefore, various interventions, including genetic modulation and the application of chemical and natural compounds, can regulate β-catenin and Dc components, potentially leading to beneficial effects in PD.

#### Modulation of Wnt Regulators

Targeting Wnt regulators, similar to other components of the Wnt pathway, has shown promise in the treatment of PD. However, in comparison with AD, therapeutic modification of the Wnt pathway via targeting its regulators is less studied in PD. The role of Dkk-1 and sFrp1/2 in the development and differentiation of midbrain DA neurons is a notable example. Besides the Dkks, sFrps have a role in controlling the non-canonical pathway and have been previously investigated for their significance in regulating midbrain DA neuron morphogenesis through the Wnt5a-associated Pcp pathway activation [85]. Targeting these regulators to restore the Wnt imbalance in PD is supported by in vitro studies demonstrating the attenuation of axonal damage in midbrain DA neurons related to Dkk-1 [161]. Finally, cyclinD1, c-myc, and transcriptional factors associated with the Wnt pathway can be modified and upregulated to benefit PD as well. Other medications and compounds, such as D1 receptor agonists, have shown effectiveness for this purpose [146, 151, 155].

## Future Directions

Wnt signaling may play a more preventive role rather than being solely therapeutic. By maintaining neuronal integrity, improving synaptic plasticity, and enhancing neurogenesis, active Wnt pathways contribute to overall brain health and increase resilience against neurodegenerative processes. As discussed, the modulation of Wnt signaling pathways is linked to reducing the accumulation of neurotoxic proteins, diminishing oxidative stress, and maintaining mitochondrial function [162]. All of these are pivotal factors in preventing the onset and progression of NDs [69, 127]. Therefore, future studies should prioritize strategies that boost Wnt signaling activity early in life or before symptoms appear, as these approaches may delay or reduce the risk of developing neurodegenerative conditions later in life.

Existing treatments of NDs focus on alleviating symptoms rather than addressing the underlying causes of the condition, primarily because the mechanisms underlying the diseases are not fully understood. The relevance and importance of Wnt pathway modification in NDs, such as AD and PD, provide a novel and promising approach to treatment. Studies have revealed that the Wnt pathway is downregulated in AD and PD and restoring Wnt activity proves beneficial. This insight paves the way for future therapeutic strategies targeting Wnt restoration in these NDs. It is noteworthy that the Wnt pathway also plays a role in other NDs, such as amyotrophic lateral sclerosis and Huntington’s disease (HD) [9]. Alterations in Wnt signaling components and downstream targets have been also observed in HD animal models and postmortem brain tissues from HD patients [163]. Studies have shown that Wnt is aberrant in endothelial cells in BBB of HD samples and downregulated other HD models [163]. Furthermore, Godin et al. have reported that mutant huntingtin disrupts β-catenin degradation by altering its binding to the Dc. Consequently, β-TrCP rescues polyQ-huntingtin-induced toxicity by promoting β-catenin degradation and targeting β-catenin homeostasis, such as with indomethacin, which holds therapeutic potential in HD [164]. In addition, the Wnt signaling pathway is also implicated in the pathophysiology of various other systemic disorders, such as cardiovascular diseases and diabetes mellitus [165–169]. NDs have complex relationships with both diabetes mellitus and cardiovascular diseases, reflecting the potential role of the Wnt signaling pathway with the systemic nature of these conditions and their risk factors [167, 169, 170]. Another issue concerning the modulation of the Wnt signaling pathway in NDs is the potential risk of cancer. The onset and progress of various cancers and NDs often entail dysregulation of overlapping molecular signaling pathways, including disruptions in the Wnt pathway [171]. Given that the Wnt signaling pathway has a crucial role in regulating the cell cycle and growth, this risk of cancer should be carefully considered when developing Wnt signaling therapies.

Hence, continued investigations are essential to understand the molecular mechanisms underlying Wnt pathway dysregulation in NDs. Simultaneously, identifying specific targets within the Wnt pathway that are aberrantly regulated in NDs is pivotal for developing targeted therapeutic interventions [150, 172, 173]. Targeting Wnt as a treatment in NDs needs more investigation to address any potential undiscovered complexity, off-targets, and adverse effects. Eventually, most of the studies have investigated the value and potential of a specific intervention to treat NDs in experimental animal models. While in vivo and in vitro experiments of a particular agent or intervention can be conducted concurrently, differences may exist between the human bodies. For instance, to lighten up the current issue, Aghaizu and colleagues showed that Dkk-2 which is a homolog of Dkk-1 is expressed differently in mouse ND models and human microglia. This study highlights a major limitation in the interpretation of data based on experimental animal models [172]. Hence, the translation of important findings from experimental studies to clinical trials is essential for evaluating the therapeutic potential of Wnt modulators in human patients. Moreover, exploring combination therapies that address various pathways involved in NDs, including the Wnt pathway, can help to enhance treatment efficacy and achieve better therapeutic outcomes. In this article, we review multiple studies that have illustrated the potential of Wnt restoration to achieve neuroprotective effects. This has been achieved by selectively targeting individual components of this system using a limited range of drugs or interventions.

## Conclusion

Due to the importance of the morbidity and mortality associated with NDs, it is necessary to develop a treatment that targets the underlying causes. The Wnt signaling pathway is closely linked to the development and maintenance of the human nervous system. In general, the Wnt pathway undergoes alterations during the progression of AD and PD. Therefore, the strategy of targeting multiple components of this cascade to restore normal physiological function or prevent pathological processes is currently under investigation. Various investigations have demonstrated diverse strategies and substances to achieve this goal, such as repurposed medications, herbal compounds, and gene editing techniques. Furthermore, the interaction between the Wnt signaling pathway and other signaling pathways is both intricate and impactful in the therapy of neurodegenerative diseases. In the end, the pathologic alteration of this pathway may be more complex in other NDs, leading to unforeseen or unintended consequences if targeted.

## Data Availability

No datasets were generated or analysed during the current study.
